# Lessons From Early COVID-19: Associations With Undergraduate Students’ Academic Performance, Social Life, and Mental Health in the United States

**DOI:** 10.3389/ijph.2022.1604806

**Published:** 2022-12-12

**Authors:** Joseph P. Nano, Mina H. Ghaly, Wen Fan

**Affiliations:** ^1^ Department of Psychology and Neuroscience, Morrissey College of Arts and Sciences, Boston College, Chestnut Hill, MA, United States; ^2^ Department of Sociology, Morrissey College of Arts and Sciences, Boston College, Chestnut Hill, MA, United States

**Keywords:** anxiety, mental health, COVID-19, depression, stress, DASS-21, undergraduate students

## Abstract

**Objectives:** This study aims to explore the influence of COVID-19 on undergraduate students’ academic performance, social life, and mental health during the pandemic’s early stage, and evaluate potential correlates of stress, anxiety, and depression in relation to COVID-19.

**Methods:** Participant data was collected as part of a survey that consisted of demographic questions, a DASS-21 questionnaire, and an open-ended question. The final sample consisted of 1077 full-time students in the United States.

**Results:** 19%, 20%, and 28% of participants met the cutoff for “severe” and “extremely severe” levels of stress, anxiety, and depression according to DASS-21. During COVID-19, a significant increase in hours of sleep, and decrease in hours spent on extracurriculars and studying were observed. While talking to family was significantly associated with stress, anxiety, and depression, engaging in hobbies was only associated with depression.

**Conclusion:** With the continued spread of COVID-19, it is critical for universities to adapt to the mental health needs of their students. Future institutional advancements should create treatment programs to ensure better academic and social outcomes.

## Introduction

In March 2020, the World Health Organization officially declared the rapidly spreading coronavirus outbreak a pandemic [1]. The pandemic has since reshaped almost every facet of modern society. Many schools and universities across the United States closed from March 2020 through the end of the Spring 2020 semester. Consequently, students living in university dormitories were required to return to tumultuous living conditions that likely detracted from learning. Educators were asked to revise, and in some cases completely revamp course standards, expectations, and assessments, all within a matter of weeks.

Despite limited evidence regarding the implications of transitioning to online learning in the context of COVID-19, previous studies have shown that the transition to postsecondary education is itself a source of anxiety [2], stress [3], and depression [2, 4]. This transition can bring about feelings of worthlessness, appetite disturbances, and issues with concentration, all of which adversely affect students’ capability to perform well in demanding environments [5]. Moreover, during the undergraduate years, students are not only immersed in higher education but are also transitioning into other critical social roles [6]. Young adults in this age range, therefore, are forced to deal with identity exploration and adjustment to university life.

Academics are exceptionally fundamental to the life and health of undergraduate students. The amount of time spent studying as well as concerns about examinations have been shown to lead to heightened immune and stress responses [7]. Therefore, coping mechanisms and social support to reduce stress are crucial, as effective coping strategies can potentially ameliorate stress reactivity [8]. In particular, understanding how the learning experience was for undergraduate students is important as online learning will likely be the primary method of instruction during future university closures. Some research has been done to assess early pandemic-related responses associated with undergraduate students’ academic work [9, 10] and mental health [11] in the United States.

Coinciding with the ever-demanding academic burden, mental health among undergraduate students represents an important and growing public health concern [12]. It was found that 12%–18% of college students suffer from a diagnosable mental illness [13]. A mental health crisis may take its toll years after the course of the COVID-19 pandemic [14]. Thus, it is important to investigate the potential factors that have adversely affected students during the pandemic.

Literature to-date is limited on commentary with regards to the effects of online learning on studying quality among undergraduates during the pandemic. The abrupt transition to online learning exploited time better spent in clinical training and internet subscription costs impeded access to effective learning for students studying at home, as did tending to family [15]. A poor internet connection was found to be a leading barrier to online learning [16].

Undergraduates are one of the most sleep-deprived age groups in the United States [17]. Studies that have investigated sleep reported significantly worsened insomnia among students during the pandemic [18, 19]. Specifically, a large cross-sectional study found a marked incidence of insomnia among college-aged students during the lockdown period [20]. Deteriorating sleep quality correlated with depressive symptoms [21].

While previous studies have commented on particular dimensions of the pandemic relating to issues of depression and anxiety [22], there remains a pressing concern to determine precisely which aspects of students’ daily life had been affected. Thus, the aim of the present study was to analyze specific correlates of stress, anxiety, and depression among undergraduate students as they relate to the most intimate issues of the college student’s demanding lifestyle. In particular, we aimed to analyze data from a large sample of students using the Depression, Anxiety, and Stress Scale (DASS-21) to approximate emotional valence. Further questions offered to students in a survey were meant to gauge whether changes in hours spent on extracurricular activities, studying, and sleep before and during the pandemic were related to observed DASS-21 results. This study also aimed to provide a better understanding about COVID-19’s influence in the context of full-time undergraduate students’ academic performance, social life, and mental health in the United States.

## Methods

### Participants

Participants were undergraduate students at a private university, public university, or community college in the United States. To be included, participants had to be at least 18 years old and enrolled as full-time students as part of class years 2020, 2021, 2022, or 2023. Between April and June 2020, participants were recruited through two channels. First, an email was sent to undergraduate students at Boston College. Second, participants were recruited online *via* Reddit, a social news platform and online forum.

An invitation to participate in the study was posted on over 20 university-related and survey recruitment subreddits (a subreddit is an online community with user-created threads dedicated to a specific topic). One such subreddit utilized for recruitment in this study was r/SampleSize, a community of over 40,000 users assembled for the express purpose of survey recruitment and participation [23]. Subreddits such as these have been shown to be a good source of diverse and viable participants [23] and are useful for inexpensive participant recruitment and reliable data collection [24, 25]. For the purpose of the current study, face-to-face interviews during this time had to be avoided because of the US lockdown and ongoing public health crisis. The study received 1,734 completed responses. After excluding students younger than 18 (*n* = 34), part-time students (*n* = 135), and students with incomplete responses (*n* = 488) from our analyses, our final sample size was 1,077.

### Measures

DASS (Depression, Anxiety, and Stress Scale): The online survey included the 21-item DASS-21 scale on mood and stress [26]. Based on the scores, participants were classified into “normal” (a score of 0–9 for depression, 0–7 for anxiety, and 0–14 for stress), “mild” (10–13, 8–9, 15–18), “moderate” (14–20, 10–14, 19–25), “severe” (21–27, 15–19, 26–33), and “extremely severe” (28+, 20+, 34+) categories. The purpose of these questions was to assess the severity of depression, anxiety, and stress during the COVID-19 pandemic. Previous studies have verified the validity of the DASS-21 scale as a routinely-used clinical and non-clinical self-report scale [27, 28]. One sample statement that participants were to score for depression was “I was unable to become enthusiastic about anything”; one sample statement for anxiety was “I worry about situations in which I might panic and make a fool of myself”; and one sample statement for stress was “I tended to over-react to situations”. Participants were given the option to choose between “once a week or less” (score = 0), “2–3 times a week” (= 1), “4–6 times a week” (= 2) and “7 times a week or more” (= 3). The corresponding sum of scores was used to assess the severity of depression, anxiety, and stress. Cronbach’s α for the items in this test was 0.934, indicating excellent internal consistency in the questionnaire.

COVID-19 Evaluation: The first portion of the survey consisted of a series of questions meant to measure how COVID-19 may have affected students’ social life and mental health. Students were asked about hours of studying per day (“1–2 h,” “3–5 h,” “5–8 h,” and “8+ h”), hours spent on extracurricular activities per week (participants typed in number of hours), and hours of sleep received per night (“4 h or less,” “5–7 h,” “7–8 h,” and “8+ h”) before the pandemic (e.g., “*How many hours of sleep did you get per night (BEFORE the COVID-19 pandemic)?*”) and during the pandemic (e.g., “*How many hours of sleep do you [currently] get per night?*”).

Coping Strategies: Participants were asked to select up to three ways they managed their stress. Seven response categories were given: “talk to friends,” “talk to family members,” “home workout/indoor sports,” “meditate,” “do favorite hobbies,” “walk outside,” and “other”. When participants chose “other”, they were asked to specify. Participants were provided with an open-ended question at the end of the survey to further elaborate on experiences that were not captured by previous questions.

### Procedure

Before taking the survey, all participants provided informed consent. Participants were made aware of all risks and benefits associated with the survey, confidentiality, and right to withdraw their voluntary participation at any time. The survey, which took 10 min to complete, consisted of four components: social demographics, school adjustments, DASS-21 questions, and an optional open-ended question. As an incentive for participation, participants were entered into a raffle for a chance to win a $10 Amazon gift card (15 participants were awarded a gift card). The survey was accessible online for 9 weeks (from April to June 2020). The procedure was approved by the university’s institutional review board in April 2020, ensuring the protection of human subjects in this research in compliance with US federal law.

### Statistical Analysis

Statistical analyses were conducted using R-Studio statistical software (version 1.3.959, 2009–2020 R-Studio, PBC). In the first phase of analysis, descriptive statistics were used to describe the demographics of the sample and the distribution of the three mental health outcomes among students. Next, t-tests were used to test whether moving to remote learning had an effect on stress, anxiety, and depression. Paired t-tests were used to determine differences in the hours of sleep, study, and extracurricular activities before and during the pandemic. Bivariate regression analysis was used to determine whether participants’ responses to moving classes to remote learning were associated with stress, anxiety, and depression. Regression analysis tested whether changes in the hours of sleep and study during COVID-19 were associated with stress, anxiety, and depression. Lastly, a multivariate OLS system was used to determine whether gender, having a family member who tested positive for COVID-19, number of times participants left their homes, school performance after moving to remote learning, and changes in the hours of sleep and studying were associated with stress, anxiety, and depression.

## Results

### Descriptive Statistics of the Sample

Most participants were students at public universities (70.5%), followed by those from private universities (28.5%) or community colleges (0.7%). 0.3% of participants preferred not to answer this question. Participants were categorized as students in the class of 2020 (16.6%), 2021 (26.1%), 2022 (29.1%), and 2023 (24.4%), respectively. 3.8% of participants preferred not to answer this question (Table 1). Female participants accounted for 51.4% of the sample. Slightly more than half of the participants were Caucasian (52.8%), one fourth were Asian/Pacific Islander (24.7%), about one tenth were Hispanic/Latinx (8.9%), and about 4% were African American (3.7%). 0.4% of participants self-identified as Native American. While the majority of participants were US students (94.3%), some participants were international students (4.9%) and a few participants preferred not to answer (0.8%).

**TABLE 1:** Demographic characteristics of participants (United States. 2020).

Demographic variable	Option categories	*%*
Gender	Male	70.5
	Female	28.5
Ethnicity	Caucasian	52.8
	Asian/Pacific Islander	24.7
	Hispanic	8.9
	African American	3.7
	Native American	0.4
	Other	4.3
	Prefer not to answer	5.3
University Classification	Public	70.5
	Private	28.5
	Community College	0.7
	Prefer not to answer	0.3
Class Year	2020	16.6
	2021	26.1
	2022	29.1
	2023	24.4
	Prefer not to answer	3.8
Student Region of Origin	US students	94.3
	International students	4.9
	Prefer not to answer	0.8

### DASS-21

**FIGURE 1:**
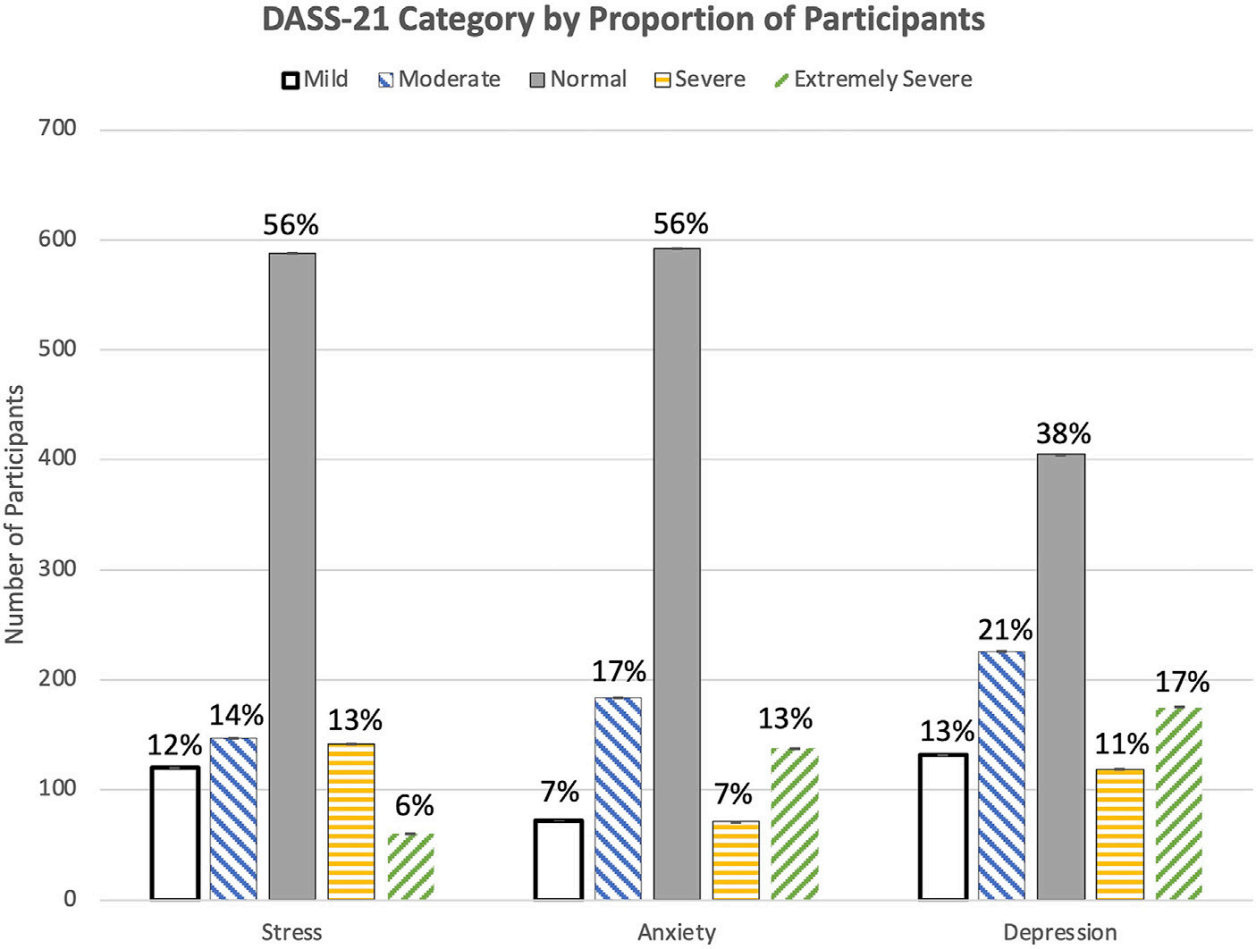
Proportion of participants whose answers on the Depression, Anxiety, and Stress Scale-21 indicated a normal, mild, moderate, severe, or extremely severe level of stress, anxiety, and depression (United States. 2020).

Using DASS-21 scores, 19%, 20%, and 28% of participants were categorized as having “severe” or “extremely severe” levels of stress, anxiety, and depression, respectively (% “severe” + % “extremely severe”) (Figure 1) (Table 2). These results indicate an increase of “severe” and “extremely severe” levels of stress, anxiety, and depression in comparison to a sample baseline, non-pandemic DASS-21 scores, with levels of stress, anxiety, and depression at 11%, 15%, and 11%, respectively [29].

**TABLE 2:** Stress, anxiety, and depression characteristics of participants (United States. 2020).

DASS-21 Category	Severity Classification	*%*
Stress	Normal (0–7)	56
	Mild (8–9)	12
	Moderate (10–14)	14
	Severe (15–19)	13
	Extremely severe (20 or higher)	6
Anxiety	Normal (0–7)	56
	Mild (8–9)	7
	Moderate (10–14)	17
	Severe (15–19)	7
	Extremely severe (20 or higher)	13
Depression	Normal (0–7)	38
	Mild (8–9)	13
	Moderate (10–14)	21
	Severe (15–19)	11
	Extremely severe (20 or higher)	17

### Bivariate Relationship Between Academic Performance, Social Life, and Mental Health

**FIGURE 2:**
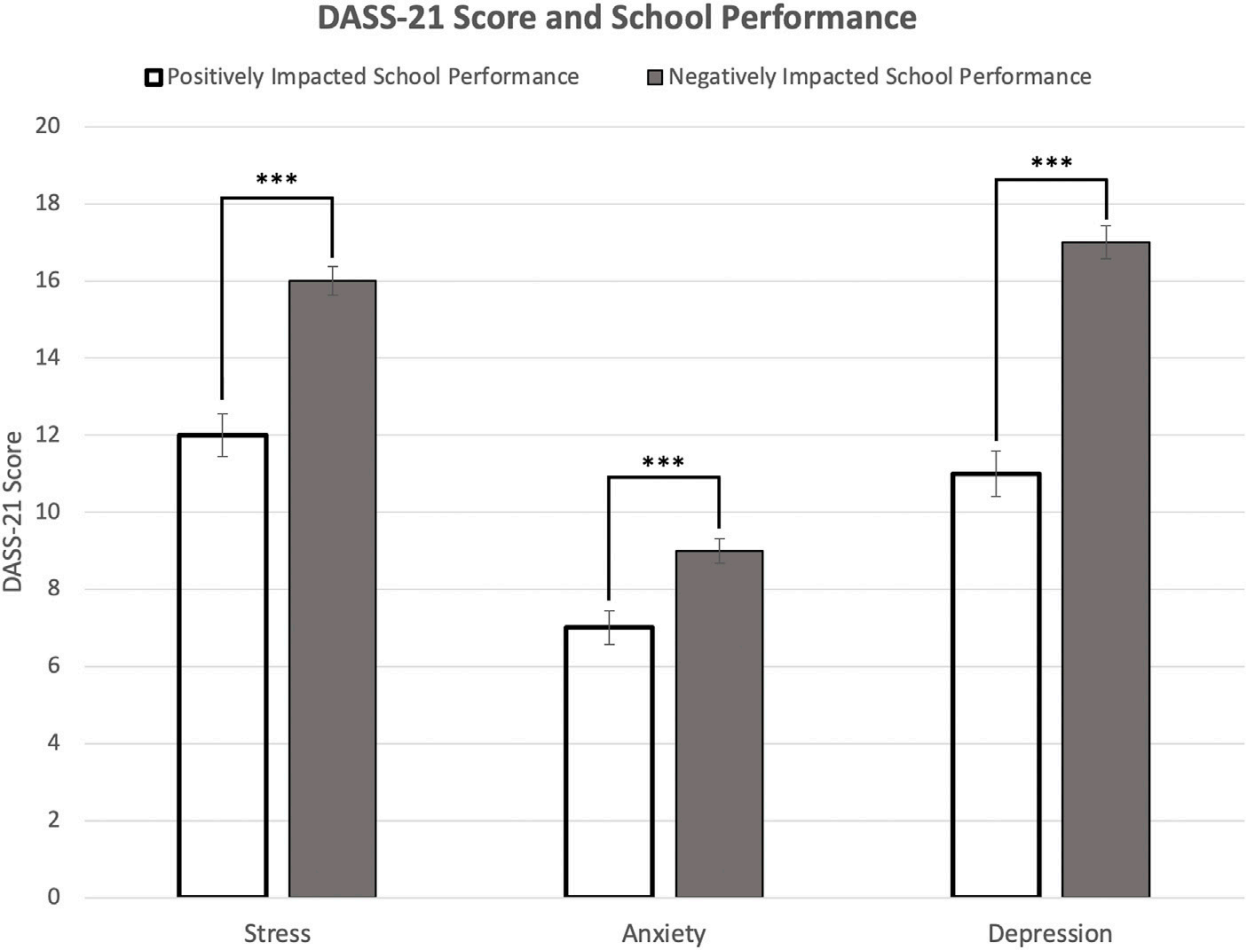
Association between stress, anxiety, and depression scores (mean Depression, Anxiety, and Stress Scale-21 score) and self-reported impact of COVID-19 on school performance (United States. 2020). Note: Error bars represent standard errors. Significance levels of Depression, Anxiety, and Stress Scale-21 scores: *p < 0.05, **p < 0.01, ***p < 0.001.

COVID-19 disrupted traditional classroom instruction and led to remote learning, as 69.3% of participants claimed that moving to remote learning had a negative impact on their school performance, while 30.7% of participants noted a positive impact of remote learning on school performance. Moreover, in terms of the association between school performance and mental health, a t-test showed that participants who claimed that remote learning had a negative impact on their school performance had significantly higher scores in stress (Figure 2, *p* < 0.001), anxiety (Figure 2, *p* < 0.001), and depression (Figure 2, *p* < 0.001), compared to peers who reported the opposite. A bivariate regression analysis further confirmed that students’ opinions about remote learning were significantly associated with stress (*p* < 0.001), anxiety (*p* < 0.001), and depression (*p* < 0.001). Participants were also asked about changes in hours spent on extracurricular activities, studying, and sleeping before and during COVID-19 comparatively. A paired two-sample t-test showed a significant increase in the hours of sleep (before COVID-19: 6.7 h; during COVID-19: 7.7 h, *p* < 0.001), a significant decrease in hours spent on extracurricular activities (before COVID-19: 9.4 h; during COVID-19: 6.2 h, *p* < 0.001), and a significant decrease in hours spent studying (before COVID-19: 4.2 h; during COVID-19: 3.6 h, *p* < 0.001).

Regression analysis was implemented to find correlates of stress, anxiety, and depression. For survey questions addressed in regard to pre-pandemic conditions, regression analysis showed that hours of sleep was associated with stress (*p* < 0.001), anxiety (*p* < 0.001), and depression (*p* < 0.001), but hours of studying was not associated with stress (*p* = 0.33), anxiety (*p* = 0.213), and depression (*p* = 0.056). However, during COVID-19, regression analysis showed that neither hours of studying nor hours of sleep were associated with stress (*p* = 0.429 and *p* = 0.678), anxiety (*p* = 0.283 and *p* = 0.506), and depression (*p* = 0.0517 and *p* = 0.665).

### Multivariate OLS Regression Models

Turning to multivariate OLS regression models, several factors were found to have significant associations with stress and anxiety. Being a male participant (*p* < 0.001), having a family member who tested positive (*p* < 0.001), leaving home three times a week (*p* < 0.05), believing that school performance was affected negatively by moving to remote learning (Figure 2, *p* < 0.001), and hours of sleep during COVID-19 (“5–6 h” *p* < 0.05, “7–8 h” *p* < 0.001, “8+ h” *p* < 0.001) were significant correlates of stress (Table 3). As for depression, being a male participant (*p* < 0.01), leaving home once a week (*p* < 0.05), believing that school performance was affected negatively by moving to remote learning (*p* < 0.001), hours spent studying (“3–5 h” *p* < 0.001, “5–8 h” *p* < 0.05), and hours of sleep during COVID-19 (“5–6 h” *p* < 0.01, “7–8 h” *p* < 0.001, “8+ h” *p* < 0.001) were significant correlates. For stress management, talking to a family member was significantly associated with stress (*p* < 0.05), anxiety (*p* < 0.05), and depression (*p* < 0.05). Engaging in favorite hobbies was only correlated with depression (*p* < 0.05).

**TABLE 3:** Multivariate Ordinary Least Squares Models for variables indicative of stress, anxiety, and depression (United States. 2020).

Variable	Stress				Anxiety				Depression			
	Sum Sq	Mean Sq	F-value	Pr (**>**F)	Sum Sq	Mean Sq	F-value	Pr (**>**F)	Sum Sq	Mean Sq	F-value	Pr (**>**F)
Graduating class	997	249.32	2.7474	0.02727*	6	1.45	0.0228	0.99899	100	25.1	0.2181	0.92839
Tested Positive to COVID-19	1837	918.58	10.1222	4.471e−05***	1751	875.32	13.7932	1.225r−06***	536	267.9	2.3325	0.09761
Gender	5234	1744.78	19.2265	4.169e−12***	2608	869.49	13.7013	9.08E−09	3164	1054.6	9.1836	5.421e−06***
Leaving Home	2081	693.81	7.6454	4.722e−05***	1747	582.46	9.1783	5.378e−06***	2958	986.1	8.5874	1.255e−05***
Impact of Remote Learning	3659	1829.39	20.159	2.672e−09***	1478	723.94	11.6441	9.982e06***	6498	3249	28.2927	1.166e−12***
Hours Study During COVID-19	715	178.71	1.9693	0.09712	523	130.77	2.1039	0.07839	2959	739.6	6.441	4.093e−05***
Hours Sleep During COVID-19	3245	1081.65	11.9192	1.152e−07***	3728	1242.82	19.9943	1.435e−12***	3836	1278.6	11.1345	3.475e−07***
Health Precautions	618	123.66	1.3627	0.23596	650	129.91	2.09	0.06444	1000	200	1.7418	0.12241
Talk to family members	6824	133.81	1.4745	0.01857*	4700	92.16	1.4826	0.01723*	8379	164.3	1.4307	0.02752*
Walk outside	1709	131.44	1.4484	0.13098	742	57.09	0.9184	0.53307	2244	172.6	1.5029	0.1098
Do favorite hobbies	1052	150.32	1.6565	0.11618	531	75.82	1.2198	0.28875	1890	270.1	2.3518	0.02200*

Note*:* *p < 0.05, **p < 0.01, ***p < 0.001.

### Sex Differences

**FIGURE 3:**
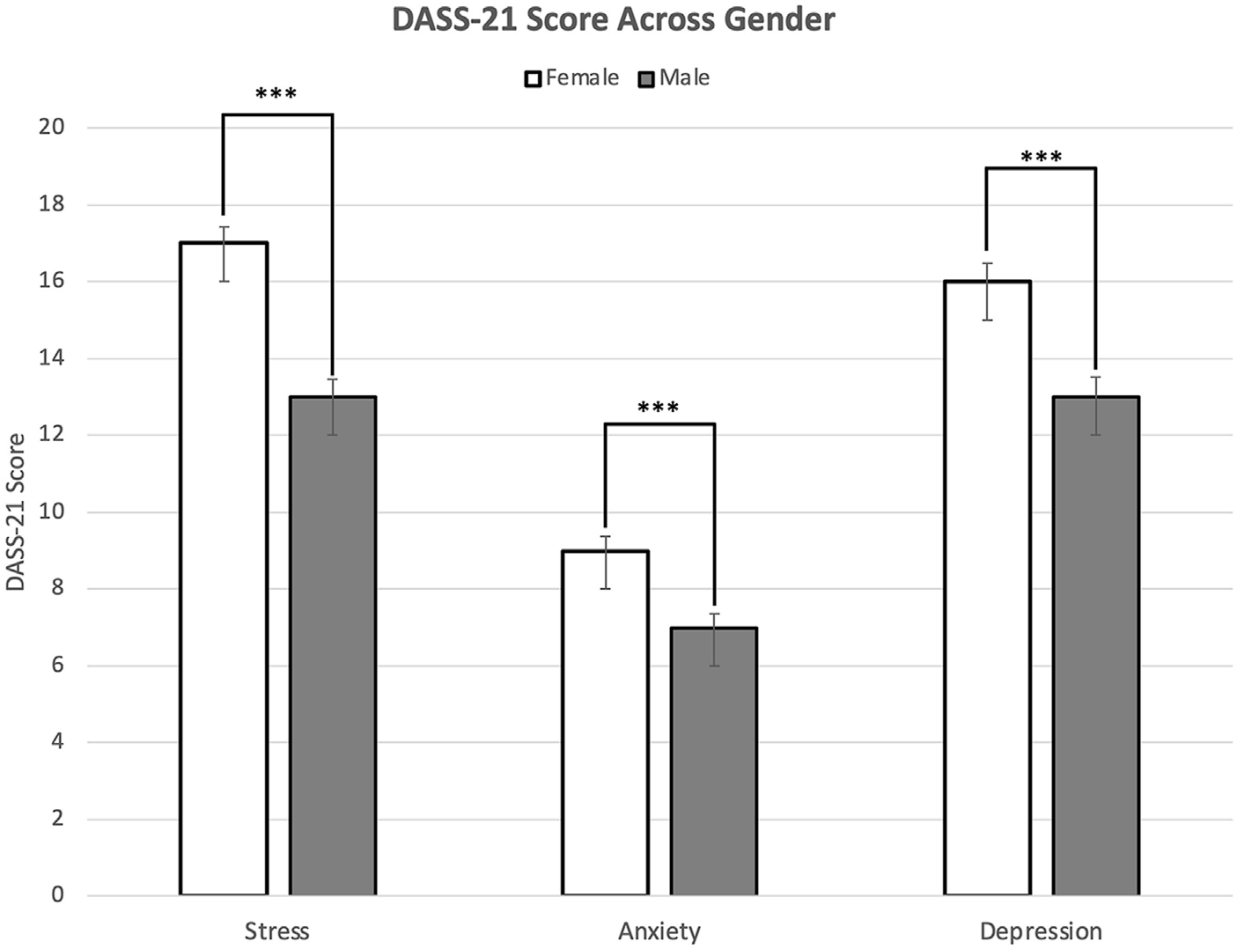
Mean Depression, Anxiety, and Stress Scale-21 scores of stress, anxiety, and depression among males and females (United States. 2020). Note: Error bars represent standard errors. Significance levels of Depression, Anxiety, and Stress Scale-21 scores: *p < 0.05, **p < 0.01, ***p < 0.001.

Female participants showed a larger percentage of severe and extremely severe levels of stress (severe; 16% and extremely severe; 7%), anxiety (8%; 15%), and depression (12%; 18%) in comparison to male participants’ stress (severe; 11% and extremely severe 3%), anxiety (5%; 10%), and depression (11%; 14%) levels (Figure 3). Chi-squared analysis showed being female was associated with severe and extremely severe levels of stress (x-squared = 30.497, df = 4, *p* < 0.001), anxiety (x-squared = 19.501, df = 4, *p* < 0.001), and depression (x-squared = 17.59, df = 4, *p* < 0.01). Two-sample t-test showed that female participants have a higher score in stress (*p* < 0.001), anxiety (*p* < 0.001), and depression (*p* < 0.001) in comparison to male participants. Regression analysis showed that gender is associated with stress (*p* < 0.001), anxiety (*p* < 0.001), and depression (*p* < 0.001).

### Stress Management

Recall that the survey asked participants to choose up to three ways they managed their stress during the COVID-19 pandemic. Almost half (47.9%) of participants reported that they talked to family members, 76% of participants engaged in their favorite hobbies, and 32.2% of participants walked outside as one of their three choices. Although the vast majority of the open-ended responses included some sentiment of stress, many individuals did have positive experiences to share. Some participants noted that conversing with significant others, in addition to immediate family, was cathartic (“I’ve been [...]reach [ing] out to my boyfriend, at the very least. These things, along with the positive experience of being around a happy family and the safety of our own home, has positively contributed to my mental wellbeing.”). In a simpler sense, some have made the most of their time while at home, with one participant noting that they were “Just staying inside and trying to learn new things.”

### Stress During COVID-19

In the open-ended question, one participant from the class of 2021 said the following of their experience during lockdown: “I have been feeling quite depressed. I feel like I have no control over my life ... I cannot plan for the future and my extracurriculars that were going to help me prepare for grad schools have been affected.” Another participant from the class of 2021 said, “Anxious and overwhelmed … I feel like I have less access to academic advising because … professors have not answered my emails. It’s been difficult to focus at home because my parents [are] working over the phone ….”

Many individuals expressed a lack of motivation to perform well on academic tasks noting severe procrastination, loss of direction, and overall dissatisfaction with the progression of the semester. One participant said, “I feel … grateful that I am healthy. However, I also have struggled to find any motivation to do my work or be active. Usually I can get things done because I look forward to having fun or relaxing on weekends, but now it is harder to get things done when it feels like that is all I am doing with nothing fun to look forward to.” Other participants found it challenging to engage with online classes. For example, one participant said, “Online learning is difficult because I feel zero engagement.”

One participant said that they felt suffocated. They said, “I want to leave the house, see new people, go to stores, but I only leave my house about 1–2 times a week.” In addition, leaving home a few times a week may have given students the opportunity to distance themselves from their families and home environment. One participant said, “My parents and I argue, and I feel like my mental health issues are having a negative effect on my family.” Another participant talked about the challenge of staying at home with family, saying, “Being at home with my family has taken a toll on my mental health. [We] do not have a great relationship, and being stuck at home has exacerbated our problems … ” One participant claimed that living temporarily away from family made them “feel great.”

## Discussion

This study looked for correlates of stress, anxiety, and depression during COVID-19. Those who reported that school performance was affected negatively by moving to remote learning exhibited significantly heightened levels of stress, as predicted by previous findings [30, 31]. The classroom environment has several advantages over online learning. Teachers, for instance, are able to receive immediate feedback on students’ understanding of key concepts [32]. Furthermore, with remote learning comes some disadvantages; problematic internet use, longer screen time, isolation, and academic pressure are all associated with psychological distress among college students [33–36].

Our results specifically showed that the frequency with which participants left their homes was significantly associated with stress and depression. Participants who left their homes every day had lower DASS-21 scores of stress, anxiety, and depression. Social relationships have been shown to give individuals a sense of purpose and greater appreciation for life, leading to overall reduced stress and bolstered mental health [37–39].

We have identified unique correlates of stress as they relate to the COVID-19 pandemic. In particular, both sleeping hours and having a family member who tested positive for COVID-19 were correlates of stress. Changes in sleep and physical activity during the pandemic were associated with symptoms of high stress [40, 41]. However, while previous studies have shown that patients with suspected COVID-19 (positive COVID-19 test result) demonstrated a significant reluctance to work [42], our study is among the first to identify the contribution of a family member’s positive test result.

This study shows a larger percentage of severe and extremely severe levels of stress, anxiety, and depression among female participants. In agreement with our results, previous studies established that the prevalence of anxiety was higher in women during the COVID-19 pandemic [43–46]. It has also been demonstrated that female students express greater concern for their future careers than do male students [47]. The dissimilarity in the levels of stress, anxiety, and depression between genders may be attributable to women seeking mental health consultation more often than men [48]. Being female was generally shown to be associated with a prominent increase in mental health problems during the pandemic [49].

This study found a significant discrepancy in hours devoted to sleep, extracurricular activities, and studying before and during the pandemic. The increase in hours of sleep could be due to heightened stress and anxiety about the pandemic during lockdown. The lockdown period had a negative impact on mental health by increasing post-traumatic stress symptoms and was associated with irregular sleep patterns [50–52]. Acute and chronic stress have been shown to perturb sleep differentially [53]. The observed decrease in hours spent on extracurricular activities during COVID-19 could be due to a fear of infection, as fear was a definite contributor to a reduction in pursuing activities and to an increase in anxiety during the pandemic [54, 55]. As a whole, engagement in regular routines was also found to lower anxiety irrespective of the kind of stressor one was exposed to [56]. Therefore, one might be able to surmise that a lack of pursuing such activities may lead to greater anxiety. In discussing the motivation to pursue meaningful work, we found that students spent less hours studying when most instruction was conducted online. Although a decrease in hours of studying could be due to changing class syllabi and adjustments to the home environment, hours of studying did not correlate with stress, anxiety, or depression. It could be that students did not feel obligated to study in an environment with less structure or in the midst of a pandemic where students took on more responsibilities at home, such as caring for siblings or supporting their own children [57, 58].

Future research should investigate current methods of mental health management for undergraduate students. The rise of telehealth and online counseling in the age of COVID-19 has provided greater opportunities for students to schedule appointments with a healthcare provider to manage mental health. Future studies might be able to explore the influence of telehealth on DASS-21 scores. Furthermore, it would be important to conduct follow up studies that investigate the impact of increased sleep on stress, anxiety, and depression during COVID-19. The adverse impact of the home environment should also be studied, specifically seeking to answer why students who left their homes less frequently experienced worsened mental health *via* higher DASS-21 scores (despite not dealing with the stresses of a daily commute, for instance).

### Limitations

Because the survey was distributed during the lockdown period, an online convenience sampling method had to have been utilized. This sampling method limited the representativeness and generalizability of the findings reported, as we were necessarily constrained to only those responses from students with access to the Internet. Thus, it is not possible to draw causal inferences due to the nature of this study. The survey also had some duplicate questions and questions that did not provide the capability to select multiple options. For example, one question asked participants about COVID-19 safety precautions taken, where the selection of multiple options could have been appropriate. The term “extracurricular” was also left to the interpretation of participants. A better survey question could have offered participants the option to define extracurricular activities. Furthermore, in addition to DASS-21, the study could have benefited from the utilization of a resilience scale to examine how certain resilience factors could have protected individuals’ mental health from COVID-19 related stress. Allowing participants to evaluate resilience may have permitted greater insight into students’ mental health and wellbeing.

### Conclusion

COVID-19 remains a credible threat to undergraduate students, beyond the acute and lingering physical effects of the virus. Students spent less time studying, complemented by the finding that the transition to remote learning hindered the majority of students’ academic experience. Students who left home more frequently may have had a greater opportunity to socialize, which lessened the stress and mental burden of lockdown. The majority of participants also stated that talking to family and engaging in favorite hobbies were beneficial for stress management. As such, these results showed that the pandemic led to significant changes in students’ academic performance, social life, and mental health.
